# Effectiveness of a pay-it-forward intervention compared with user-paid vaccination to improve influenza vaccine uptake and community engagement among children and older adults in China: a quasi-experimental pragmatic trial

**DOI:** 10.1016/S1473-3099(22)00346-2

**Published:** 2022-10

**Authors:** Dan Wu, Chenqi Jin, Khaoula Bessame, Fanny Fong-Yi Tang, Jason J Ong, Zaisheng Wang, Yewei Xie, Mark Jit, Heidi J Larson, Tracey Chantler, Leesa Lin, Wenfeng Gong, Fan Yang, Fengshi Jing, Shufang Wei, Weibin Cheng, Yi Zhou, Nina Ren, Shuhao Qiu, Jianmin Bao, Liufen Wen, Qinlu Yang, Junzhang Tian, Weiming Tang, Joseph D Tucker

**Affiliations:** a West China School of Public Health, West China Fourth Hospital, Sichuan University, Chengdu, China; b Department of Clinical Research, Faculty of Infectious and Tropical Diseases, London School of Hygiene & Tropical Medicine, London, UK; c Faculty of Epidemiology and Population Health, London School of Hygiene & Tropical Medicine, London, UK; d Faculty of Public Health and Policy, London School of Hygiene & Tropical Medicine, London, UK; e Chapel Hill Project-China, University of North Carolina, Guangzhou, China; f Shanghai Institute of Nutrition and Health, Chinese Academy of Sciences, Shanghai, China; g Li Ka Shing Faculty of Medicine, University of Hong Kong, Hong Kong Special Administrative Region, China; h Faculty of Medicine, Central Clinical School, Monash University, Melbourne, VIC, Australia; i Melbourne Sexual Health Centre, Alfred Health, Melbourne, VIC, Australia; j School of Health and Related Research, University of Sheffield, Sheffield, UK; k Duke Global Health Institute, Duke University, Durham, NC, USA; l Laboratory of Data Discovery for Health, Hong Kong Science Park, Hong Kong Special Administrative Region, China; m Beijing Representative Office, Bill & Melinda Gates Foundation, Beijing, China; n Institute of Population Research, Peking University, Beijing, China; o Guangdong Secondary Provincial General Hospital, Guangzhou, China; p Zhuhai Center for Disease Control and Prevention, Zhuhai, China; q Faculty of Medicine, Macau University of Science and Technology, Macau Special Administrative Region, China; r Vaccination Clinic, Yangshan Health Centre, Qingyuan, China; s Fenghuang Community Health Service Centre, Zengcheng, China; t Xinhua Community Health Service Centre, Guangzhou, China; u Community Health Centre, Guangzhou, China; v Institute for Global Health and Infectious Diseases, School of Medicine, University of North Carolina, Chapel Hill, NC, USA

## Abstract

**Background:**

China has low seasonal influenza vaccination rates among priority populations. In this study, we aimed to evaluate a pay-it-forward strategy to increase influenza vaccine uptake in rural, suburban, and urban settings in China.

**Methods:**

We performed a quasi-experimental pragmatic trial to examine the effectiveness of a pay-it-forward intervention (a free influenza vaccine and an opportunity to donate financially to support vaccination of other individuals) to increase influenza vaccine uptake compared with standard-of-care user-paid vaccination among children (aged between 6 months and 8 years) and older people (≥60 years) in China. Recruitment took place in the standard-of-care group until the expected sample size was reached and then in the pay-it-forward group in primary care clinics from a rural site (Yangshan), a suburban site (Zengcheng), and an urban site (Tianhe). Participants were introduced to the influenza vaccine by project staff using a pamphlet about influenza vaccination and were either asked to pay out-of-pocket at the standard market price (US$8·5–23·2; standard-of-care group) or to donate any amount anonymously (pay-it-forward group). Participants had to be eligible to receive an influenza vaccine and to have not received an influenza vaccine in the past year. The primary outcome was vaccine uptake. Secondary outcomes were vaccine confidence and costs (from the health-care provider perspective). Regression methods compared influenza vaccine uptake and vaccine confidence between the two groups. This trial is registered with ChiCTR, ChiCTR2000040048.

**Findings:**

From Sept 21, 2020, to March 3, 2021, 300 enrolees were recruited from patients visiting three primary care clinics. 55 (37%) of 150 people in the standard-of-care group (40 [53%] of 75 children and 15 [20%] of 75 older adults) and 111 (74%) of 150 in the pay-it-forward group (66 [88%] of 75 children and 45 [60%] of 75 older adults) received an influenza vaccine. People in the pay-it-forward group were more likely to receive an influenza vaccine compared with those in the standard-of-care group (adjusted odds ratio [aOR] 6·7 [95% CI 2·7–16·6] among children and 5·0 [2·3–10·8] among older adults). People in the pay-it-forward group had greater confidence in vaccine safety (aOR 2·2 [95% CI 1·2–3·9]), importance (3·1 [1·6–5·9]), and effectiveness (3·1 [1·7–5·7]). In the pay-it-forward group, 107 (96%) of 111 participants donated money for subsequent vaccinations. The pay-it-forward group had a lower economic cost (calculated as the cost without subtraction of donations) per person vaccinated (US$45·60) than did the standard-of-care group ($64·67).

**Interpretation:**

The pay-it-forward intervention seemed to be effective in improving influenza vaccine uptake and community engagement. Our data have implications for prosocial interventions to enhance influenza vaccine uptake in countries where influenza vaccines are available for a fee.

**Funding:**

Bill & Melinda Gates Foundation and the UK National Institute for Health Research.

## Introduction

In mainland China, an average of ten people die from influenza-related illnesses every hour.^1^ Influenza vaccination is the most effective way to prevent morbidity and mortality attributable to influenza.^2^ Influenza vaccination has been particularly important during the COVID-19 pandemic because research has suggested that it might help reduce risks of acquiring SARS-CoV-2 and reduce COVID-19 severity.^3^, ^4^ The Chinese Center for Disease Control and Prevention (CDC) guidelines recommend influenza vaccination for populations at high risk of infection and influenza-related complications, including children and older adults. However, influenza immunisation policies widely vary,^5^ and most cities in China do not provide free influenza vaccines to individuals at high risk of infection and influenza-related complications (high-risk individuals). A meta-analysis reported that 11·9% of children aged 6 months to 17 years and 21·7% of adults aged 60 years and older in China received an influenza vaccine in the most recent year with data available (2015–16 for children and 2014–15 for older adults).^6^ Low influenza vaccine uptake is common in many other low-income and middle-income countries.^7^

**Research in context**

**Evidence before this study**

The burden of influenza-attributable diseases is high in many low-income and middle-income countries (LMICs). Seasonable vaccination is the most effective prevention; however, influenza vaccine uptake is low in LMICs. Common reasons for low vaccination rates include little public funding and awareness, and public distrust. Most interventions to enhance vaccine uptake are focused on high-income countries. We searched PubMed and Google Scholar for studies reporting influenza vaccination among Chinese populations that were published between database inception and March 9, 2022, with the search terms “China”, AND “influenza vaccination” OR “influenza vaccine” OR “flu vaccination” OR “flu vaccine”. No language restrictions were applied. We identified a systematic review published in 2018 that suggested that only 7.0% of the general population, 11·9% of children aged 6 months to 17 years, and 21·7% of adults aged 60 years and older received an influenza vaccine in the most recent year with data available (2016–17 for the general population, 2015–16 for children, and 2014–15 for older adults) in China. Most evaluation studies were observational and focused on places that were implementing free or reimbursed influenza vaccination policies. We found a protocol paper describing an ongoing educational intervention aiming to improve willingness to vaccinate and uptake among older adults. We also found a randomised controlled trial using text message reminders to mothers, but the trial found no effect on vaccine uptake among children. A cluster randomised trial evaluating the effect of a comprehensive educational intervention among patients with diabetes reported an influenza vaccine uptake rate of 45·8% in the intervention group and 27·4% in the control group. We did not find any interventions involving community engagement, and none of the studies assessed vaccine trust. We found one randomised controlled trial evaluating the effect of a pay-it-forward intervention in improving gonorrhoea and chlamydia test uptake among sexual minority groups, but not in vaccine services.

**Added value of this study**

This study evaluated a pay-it-forward innovation for influenza vaccination among children and older people in China. We found that a pay-it-forward intervention increased vaccine uptake and had lower costs per person vaccinated compared with the standard-of-care user-paid vaccination. The pay-it-forward model also involved and generated community engagement and substantially enhanced participant confidence in vaccine importance, safety, and effectiveness.

**Implications of all the available evidence**

Our pay-it-forward intervention might be a promising model for vaccine service delivery that could help enhance public confidence and vaccine uptake among priority populations in places where free or subsidised vaccine services are unavailable and help transition from out-of-pocket payments to government-funded influenza vaccination programmes. The intervention might also have implications for prosocial interventions to address public distrust and hesitancy in vaccine services.

There are several reasons for low uptake of influenza vaccination in China.^6^, ^8^ First, most people in China are unaware of influenza vaccination, and many people are unsure about vaccine safety and effectiveness.^9^ Second, there is minimal community engagement in vaccinations.^10^ Community engagement is the process of working collaboratively with groups of people affiliated by proximity, interests, or situations with respect to issues affecting their wellbeing.^11^ Despite a strong rationale for community engagement,^12^ most programmes aiming to increase vaccination have used educational messages,^13^, ^14^, ^15^ and none have engaged the public regarding influenza vaccinations.^16^, ^17^ Third, there is limited public funding to support influenza vaccination among high-risk populations. Observational studies have suggested that free or reimbursed vaccine policies can improve vaccine uptake,^18^ but influenza vaccination is mostly not covered by mandatory health insurance schemes in China. As a result, most people have to pay US$8·5–23·2 out-of-pocket to be vaccinated.^19^ Innovative strategies are needed to improve influenza vaccine uptake.

Pay-it-forward interventions have one individual receive a gift or free service and then invite them to give a gift to another person.^20^ Our previous pay-it-forward studies focused on increasing testing for sexually transmitted infections among sexual minority populations in sexual health clinics. The pay-it-forward group had a chlamydia and gonorrhoea dual test uptake of 56% compared with 18% in the standard-of-care group, where participants had to pay out-of-pocket.^21^, ^22^ More than 90% of participants in the pay-it-forward group donated to the rolling finance pool, and qualitative data showed that trust in health services improved among participants in the pay-it-forward group.^23^ However, pay-it-forward interventions have not been examined for increasing vaccination services uptake in community-based primary care facilities in the public sector.

This quasi-experimental pragmatic trial assessed the effectiveness of a pay-it-forward intervention to increase influenza vaccination uptake at three study sites among children (aged between 6 months and 8 years) and older adults (aged ≥60 years) in comparison with the current standard of care (user-paid vaccination) in Guangdong, China.

## Methods

### Study design and participants

Guangdong is a subtropical province in southern China with over 120 million people and influenza is prevalent throughout the year.^25^ In this quasi-experimental pragmatic trial, we selected three study sites to reflect varying economic conditions—higher-income, middle-income, and lower-income level regions. These three study sites were a rural site (Yangshan; lower income), a suburban site (Zengcheng; middle income), and an urban site (Tianhe; higher income). Clinics were selected because they had sufficient influenza vaccines in stock and medical staff (nurses and doctors) who were familiar with influenza vaccination. All these clinics were primary care clinics that provided vaccination services for local residents in the neighbourhood. The scope of essential primary care services was similar across the three clinics (ie, common medical conditions, chronic diseases, vaccination services, and other preventative public health tasks). Most participants were regular attendees to the clinics. Influenza vaccination requires a fee in most cities and rural parts of China. There are some pilots in urban areas that provide free influenza vaccines. All three of the sites in this study still had fees associated with influenza vaccination for local residents.

The inclusion criteria for this study differed by age group and were created according to China's national influenza vaccine guidelines.^25^ Eligibility criteria were aged between 6 months and 8 years (children) or 60 years or older (older adults), no acute moderate or severe illnesses, eligible to receive an influenza vaccine on the basis of clinical evaluation from a physician, has a legal guardian (children) or capable of making informed decisions (older adults), consents to participate in the study, and has not received an influenza vaccine in the past year. We only allowed one person per family to join the study. All eligible children and older adults presenting to the study sites were invited to participate by medical staff involved in the study. We obtained consent from guardians of children and older adults via an online consent form before they started filling in the survey.

Ethical approval for this study was obtained from the institutional review boards at the London School of Hygiene & Tropical Medicine (London, UK) and the Zhuhai Center for Disease Control (Zhuhai, China).

### Participant allocation

Because community health-care workers had heavy workload due to COVID-19, we anticipated limited organisational willingness and capacity to help implement administratively demanding randomisation and recruitment for a randomised trial. Therefore, we adopted a non-random approach for this trial. Recruited participants were chronologically allocated into the specified study groups (appendix p 7).

Each study site recruited all study groups. At each site, participants were first recruited into the standard-of-care group, followed by the pay-it-forward group, and finally the free-service group. Influenza vaccine services are usually available in China from Sept 1 to April 30. Influenza vaccine availability is idiosyncratic at specific study sites because of supply chain problems in local settings. Despite discussions with health authorities and vaccine manufacturers, study sites encountered lapses in supply. The time needed to recruit each study group was related to the availability of vaccines and the number of people willing to participate.

### Procedures

This study consisted of three stages: cocreation^26^ of the pay-it-forward intervention and engagement strategies with stakeholders (a Chinese vaccine expert, a communication specialist, a public health researcher, and a infectious disease physician with children) during a 3 day hackathon (Nov 4–6, 2019),^27^ a sprint event that brings together diverse individuals to collectively solve a problem, in Dar Es Salaam, Tanzania; a feasibility pilot study to inform the recruitment process and sample size calculations; and a quasi-experimental pragmatic trial to evaluate the effectiveness of the intervention. Participants of the hackathon included potential end users, public health practitioners, health innovators, communication experts, and vaccine experts. The hackathon mapped out the following key components of the study: key stakeholders, potential user journeys, behavioural mechanisms (appendix p 1), donation strategies (appendix pp 1–2), and engagement strategies (appendix pp 2–6). These key components were later contextualised into the local settings in China and iteratively adapted by involving local stakeholders and experts (ie, community representatives, community-based vaccination clinic staff, pharmaceutical producers, vaccine research expert, and communication specialists). The feasibility pilot study was done at the rural study site from Dec 9, to April 29, 2020, during COVID-19 restrictions. In the pilot feasibility trial, 40 (91%) of 44 participants in the pay-it-forward group and 13 (23%) of 57 participants in the standard-of-care group received an influenza vaccine.

Data collection for the quasi-experimental pragmatic trial began on Sep 21, 2020, after confirmation of vaccine availability by local study sites, and completed on March 3, 2021, when China started the COVID-19 vaccine roll-out nationwide. Participants recruited in the standard-of-care group were briefly introduced to the influenza vaccine by project staff using a pamphlet about influenza and influenza vaccination (appendix p 7). Participants were then asked if they were willing to pay out-of-pocket at the standard market price (US$8·5–23·2, depending on the market price of vaccines provided at the clinic) to receive an influenza vaccination. Participants who agreed to pay were screened for vaccination eligibility, and those without any contraindications received the vaccine.

Participants recruited in the pay-it-forward group were provided with the same introductory pamphlet about influenza and influenza vaccination as used in the standard-of-care group. Project staff then explained the pay-it-forward programme (appendix p 8), including its purpose, the opportunity to receive one dose of influenza vaccination for free, and the opportunity to donate money towards someone else's vaccine dose and write postcard messages. Participants were told that the normal cost of an influenza vaccine, including administration fees, was ¥56 (US$8·5) for children and ¥153 ($23·2) for adults and that previous participants had donated money to cover the costs of their vaccine and had created handwritten postcard messages for them.

If participants in the pay-it-forward group decided to receive the vaccine, they were asked before vaccination whether they were willing to donate any amount of money into a pool of funds to support subsequent participants in receiving the same vaccine. They were assured that the donation was entirely voluntary, that any donation amount was acceptable, and that donation would not affect whether they received a vaccination or subsequent care. They were also invited to write anonymous postcard messages for future participants. A donation collection box was provided onsite for those who preferred to donate cash. A QR code using WeChat was provided to those who chose to make online donations. Donations were anonymous and project staff were unaware of the donation amount. Donations were used to support the vaccination of subsequent participants, and aggregated data on donation amounts were made publicly available on the website and WeChat newsletter of Social Entrepreneurship to Spur Health (a research hub in the UNICEF, UNDP, World Bank, and WHO Special Programme for Research and Training in Tropical Diseases Social Innovation in Health Initiative). COVID-19 conditions (eg, physical distancing) prevented participants from creating handwritten postcards during some periods of the trial. Participants in the free-service group were invited to participate using the same introductory pamphlet and were provided with free influenza vaccination. They did not receive any community-created messages about the pay-it-forward programme.

Participation was voluntary and anonymous. After introducing the intervention before vaccination, all participants were asked to complete a short, self-administered online questionnaire to collect information about sociodemographic characteristics and attitudes towards influenza vaccines (appendix pp 20–27). Vaccine confidence in importance, safety, and effectiveness were measured using survey items adapted to assess influenza vaccine confidence in China.^28^, ^29^ Participants who had difficulty reading the questionnaire were assisted by the project and health-care staff onsite. A small gift worth around ¥10 (US$1·5) was given to each participant after completing the questionnaire survey. Administrative and survey data were linked using identification numbers.

After receiving the vaccination, participants in both groups stayed in the waiting room for 30 mins and severe adverse effects were observed clinical staff.

### Study outcomes

The primary outcome was influenza vaccine uptake ascertained by administrative records. Secondary outcomes were self-reported vaccine confidence (defined as public trust in the vaccine safety, importance, and effectiveness^30^) and cost of each strategy.

### Data analysis

Given the differences in sociodemographic backgrounds and determinants of influenza vaccination between children and older adults, we stratified sample size calculations by age groups. On the basis of our pilot data, we estimated that vaccine uptake would be 30% in the standard-of-care group and 80% in the pay-it-forward group. Thus, a sample size of 100 participants (50 participants in the control group and 50 in the intervention group) for each age group would give 90% power to test that the pay-it-forward invention is superior to the standard-of-care intervention in promoting vaccination uptake, with a margin of 10% and a significance level of 0·025. We increased the sample size by 50% to allow for secondary analyses, resulting in a sample size of 75 participants for each age group in each group.

In addition to the primary comparison (pay it forward *vs* standard of care), we implemented an exploratory group (n=150, 75 children and 75 older adults), in which participants were offered free influenza vaccination without any community engagement. We included a free vaccine group because it provided an opportunity to compare the cost of pay-it-forward interventions with free-service provision (appendix pp 13–19); however, the study was not powered to assess the difference in vaccine uptake between pay-it-forward and free-service groups.

Descriptive analyses were done to summarise sociodemographic and behavioural characteristics, participation rate, and vaccination rate. We used a χ^2^ test to investigate differences in vaccination uptake between the standard-of-care and pay-it-forward groups. We ran multivariable logistic regression models to examine the association between outcomes (vaccine uptake and vaccine confidence) and interventions (standard-of-care and pay-it-forward groups) after adjusting for age, sex, study site, education level, occupation, income, and marital status. We selected these potential confounders on the basis of previous reports of factors associated with influenza vaccination and detected differences in sociodemographic backgrounds of the participants between the three study sites. Data for the vaccine uptake were complete and all participants were included in analyses. Participants with missing data for the vaccine confidence variables were excluded from analyses of confidence in vaccine safety, importance, and effectiveness outcomes. We summarised the participants' donations in the pay-it-forward group and compared proportions of participants between rural, suburban, and urban sites who contributed US$7·59 (close to a child vaccine cost) or more.

We evaluated the costs of the standard-of-care and pay-it-forward interventions using a microcosting approach and reported costs in US$(2020). The costs of implementing each group were estimated using invoices, onsite staff's self-reporting of the wages of health-care workers, and estimated opportunity costs (ie, the estimated total time spent on pay-it-forward-related activities) of community staff's time (appendix pp 13–19). Additional costs in the pay-it-forward group related to volunteer time and the recruitment and donation processes. We excluded research-related costs. Financial costs were obtained by subtracting donation contributions from the total economic cost. The analysis was done from the health-care provider's perspective (Guangdong Department of Health). We reported the total economic and financial costs for each group and the cost per person vaccinated.

All data were analysed using SPSS (version 25) and Strata (version 17).

### Role of the funding source

One vaccine research expert (WG) from the Bill & Melinda Gates Foundation served as an adviser to help intervention design and interpretation of the data. The UK National Institute for Health Research had no role in study design, data collection, data analysis, data interpretation, or writing of the report.

## Results

In total, 184 children's caregivers and 182 older adults were approached at the three study sites (figure). 41 people declined to participate before being assigned a treatment group and 25 had received the influenza vaccine in the past year. In total, 150 people were enrolled in the standard-of-care group and 150 in the pay-it-forward group (table 1). All 300 responses were screened for completeness and were included in the final statistical analyses.

**Figure:**
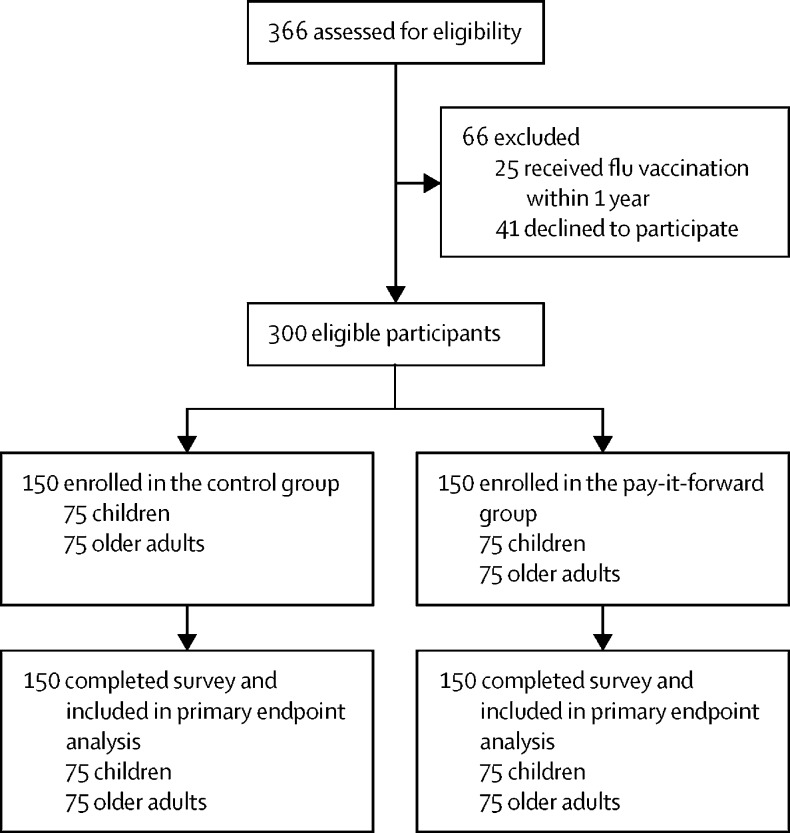
Trial profile

**Table 1:** Characteristics of recruited child caregivers and older adults (≥60 years)

		**Child caregivers**			**Older adults**		
		Standard-of-care (n=75)	Pay-it-forward (n=75)	p value	Standard-of-care (n=75)	Pay-it-forward (n=75)	p value
Study site		..	..	..	..	..	..
	Rural Yangshan	25 (33%)	25 (33%)	..	25 (33%)	25 (33%)	..
	Suburban Zengcheng	25 (33%)	25 (33%)	..	25 (33%)	25 (33%)	..
	Urban Tianhe	25 (33%)	25 (33%)	..	25 (33%)	25 (33%)	..
Age, years		35·91 (10·3)	36·71 (9·7)	0·62	69·53 (6·4)	66·52 (6·7)	0·0060
Sex		..	..	0·84	..	..	0·22
	Male	17 (23%)	16 (21%)		20 (27%)	27 (36%)	
	Female	58 (77%)	59 (79%)	..	55 (73%)	48 (64%)	..
Education		..	..	<0·0001	..	..	0·19
	Elementary school or below	8 (11%)	4 (5%)	..	33 (44%)	26 (35%)	..
	Middle school	45 (60%)	26 (35%)	..	31 (41%)	42 (56%)	..
	Undergraduate or higher	22 (29%)	45 (60%)	..	11 (15%)	7 (9%)	..
Occupation		..	..	0·98	..	..	0·64
	Unemployed	20 (27%)	21 (28%)	..	53 (71%)	58 (77%)	..
	Farmer	1 (1%)	1 (1%)	..	19 (25%)	15 (20%)	..
	Employed	54 (72%)	53 (71%)	..	3 (4%)	2 (3%)	..
Annual income (US$)		..	..	0·79	..	..	0·42
	0 to 1860	19 (25%)	23 (31%)	..	38 (51%)	28 (37%)	..
	>1860 to 9300	22 (29%)	24 (32%)	..	29 (39%)	36 (48%)	..
	>9300 to 18 600	20 (27%)	16 (21%)	..	7 (9%)	10 (13%)	..
	>18 600	14 (19%)	12 (16%)	..	1 (1%)	1 (1%)	..
Marital status		..	..	1·00	..	..	1·00
	Single, divorced, separated, or widowed	4 (5%)	4 (5%)	..	20 (27%)	20 (27%)	..
	Married or living with a partner	71 (95%)	71 (95%)	..	55 (73%)	55 (73%)	..

Data are n (%) or mean (SD).

55 (37%) of 150 people in the standard-of-care group (40 [53%] of 75 children and 15 [20%] of 75 older adults) and 111 (74%) of 150 in the pay-it-forward group (66 [88%] of 75 children and 45 [60%] of 75 older adults) received an influenza vaccine (appendix p 11). Adjusted odds ratios (aOR) in table 2 suggest that people in the pay-it-forward group were more likely to receive the vaccine than people in the standard-of-care group among children (aOR 6·7 [95% CI 2·7–16·6]) and older adults (5·0 [2·3–10·8]).

**Table 2:** Multivariable logistic regression to compare influenza vaccine uptake rates between the standard-of-care and pay-it-forward groups

	**Children (N=150)**		**Older adults (N=150)**	
	Crude OR (95% CI)	Adjusted OR (95% CI)	Crude OR (95% CI)	Adjusted OR (95% CI)
Standard-of-care (reference)	..	..	..	..
Pay-it-forward	6·4 (2·8–14·7)	6·7 (2·7–16·6)	6·00 (2·9–12·5)	5·0 (2·3–10·8)
p value	p<0·0001	p<0·0001	p<0·0001	p<0·0001

For adjusted OR, the model adjusted for age, sex, study site, education level, occupation, income, and marital status. OR=odds ratio.

High proportions of participants reported being confident about vaccine safety (215 [75%] of 285; 15 [5%] of 300 participants were excluded as they had missing data for vaccine confidence variables), importance (224 [79%]), and effectiveness (210 [74%]). Compared with child caregivers and older adults in the standard-of-care group, people in the pay-it-forward group were more likely to be confident about influenza vaccine safety (83% *vs* 67%; aOR 2·2 [95% CI 1·2–3·9]), vaccine importance (88% *vs* 69%; 3·1 [1·6–5.9]), and vaccine effectiveness (85% *vs* 62%; 3·1 [1·7–5·7]; table 3). We observed no serious adverse events adverse events among all participants.

**Table 3:** Multivariable logistic regression to compare vaccine confidence between standard-of-care and pay-it-forward groups (N=285)

	**Confidence in safety**			**Confidence in importance**			**Confidence in effectiveness**		
	Total (%), n	Crude OR (95% CI)	Adjusted OR (95% CI)	Total (%), n	Crude OR (95% CI)	Adjusted OR (95% CI)	Total (%), n	Crude OR (95% CI)	Adjusted OR (95% CI)
Standard-of-care (reference; N=141)	95 (67%)	..	..	97 (69%)	..	..	88 (62%)	..	..
Pay-it-forward (N=144)	120 (83%)	2·4 (1·4–4·2)	2·2 (1·2–3·9)	127 (88%)	3·4 (1·8–6·3)	3·1 (1·6–5·9)	122 (85%)	3·3 (1·9–5·9)	3·1 (1·7–5·7)
p value	..	0·0020	0·010	..	<0·0001	<0·0001	..	<0·0001	<0·0001

For adjusted OR, the model adjusted for age, sex, study site, education level, occupation, income, and marital status. OR=odds ratio.

Regarding participant contributions and engagement in the pay-it-forward group, 107 (96%) of 111 participants who received the influenza vaccine donated money, with a total contribution of US$604·46 (covering 31·6% of vaccination costs). The median donation was $4·55 (IQR 1·52–7·59); the distribution of donations by study site is shown in the appendix (p 9). Only 12 (30%) of 40 people who donated in the rural site contributed $7·59 (¥50; close to the price of a child vaccine) or more, compared with 26 (62%) of 42 people who donated in the suburban site and ten (40%) of 25 people who donated in the urban site (appendix p 12). Of those who donated, 19 (32%) of 60 people given the opportunity to write postcards wrote messages for subsequent participants.

The total financial cost for the health-care provider of implementing an influenza vaccination intervention for participants was US$2725 for the standard-of-care group and $4477 for the pay-it-forward group. The financial cost per person vaccinated was $49·55 in the standard-of-care group and $40·33 in the pay-it-forward group. The economic cost of implementing an influenza vaccination intervention for children and older adults was $3557 for standard-of-care and $5062 for pay-it-forward groups. The economic cost per person vaccinated was $64·67 for the standard-of-care and $45·60 for the pay-it-forward groups. The financial and economic costs per person vaccinated in the pay-it-forward group were close to those in the free vaccine group ($40·92 for both financial and economic costs). We provide a more detailed breakdown of cost estimation in the appendix (p 13–19). In brief, for the pay-it-forward group, 52% of the total economic cost was related to recurrent costs, 46% to fixed costs, and 2% to start-up costs (appendix p 19). In contrast, for the standard-of-care group, 34% of the total economic cost was related to recurrent costs, 65% to fixed costs, and 1% to start-up costs.

## Discussion

Our study contributes to the literature by assessing the effectiveness of a social innovation intervention using a quasi-experimental design, developing new methods for public influenza vaccination engagement, and enhancing vaccine uptake. Our data suggest that the pay-it-forward strategy might increase influenza vaccine uptake among high-risk individuals compared with a self-pay strategy for vaccination. This strategy increased vaccine uptake compared with the standard of care, elicited financial contributions, and was correlated with vaccine confidence.

The finding that children and older adults who took part in the pay-it-forward intervention had higher influenza vaccine uptake than those in the self-pay intervention is consistent with previous intervention studies using pay-it-forward interventions to improve health services uptake.^21^, ^22^ The vaccination rate in the pay-it-forward group was also higher than the rate in Chinese cities (47·5%^6^) in other studies where influenza vaccination was partly or fully reimbursed and in studies where educational interventions were used.^14^, ^15^ The effect of pay-it-forward interventions might be related to the reduced costs of vaccination, enhanced community engagement, vaccine confidence, or a combination of these.

We also observed that, among those enrolled in the pay-it-forward group, nearly all voluntarily donated to support another person after receiving an influenza vaccine, including those with a low annual income from a study site in a poor rural area. In addition, the pay-it-forward intervention had lower costs per person vaccinated than the standard-of-care practice. The financial cost per person vaccinated in the pay-it-forward intervention was also lower than the median cost (US$50·78) per additional enrolee vaccinated from a systematic review published in 2018.^31^ Donations collected using a pay-it-forward system can support more individuals in receiving influenza vaccine services, which could be an important social innovation for improving influenza vaccine uptake when government-funded vaccination is unavailable. Pay-it-forward interventions could help transition out-of-pocket payments to government-funded influenza vaccine programmes. Furthermore, the higher observed average donation amount in the urban and suburban areas than in the rural area suggests the possibility of creating an urban-to-rural subsidisation mechanism to support influenza vaccination in poorer areas.

Pay-it-forward interventions have additional social benefits, fostering community engagement.^10^ Community engagement is central to the success of public health programmes. Given that some engagement methods could facilitate influenza transmission,^32^ it is important to identify community engagement methods that are safe and effective. COVID-19-related measures during earlier periods of the trial prevented us inviting participants in the pay-it-forward group to write postcards and we managed to engage some of them only when these measures were eased. Engaging the community in vaccination services through cultivating kindness and reciprocity might also strengthen community solidarity and increase confidence in vaccine services.^23^, ^33^

The study has several limitations. First, our study was implemented after COVID-19 lockdowns had ended, but all sites were heavily focused on COVID-19 prevention and related activities, which caused some delays in recruitment. There were small outbreaks in Guangzhou during the study period (potentially leading to local residents and health staff may be more cautious about COVID-19 and preventive measures), but residents in our study area were able to live their lives with minimal non-pharmaceutical interventions related to COVID-19 prevention. This study showed the feasibility of implementing pay-it-forward interventions during emergency responses. Additionally, we anticipated that there might be an increase in acceptance of influenza vaccination^34^ and uptake.^35^ However, the effect of the COVID-19 pandemic is expected to be similar across the two groups. COVID-19 vaccine roll-out in China started after our recruitment ceased and the COVID-19 vaccine roll-out unlikely affected the study outcomes. Second, we examined people from only three sites with varying levels of economic conditions and the sites were selected on the basis of the availability of vaccines, organisational willingness, and capacity to collaborate. Selection biases caused by convenience sampling at the three study sites are possible. However, all of our sites had a high influenza prevalence, were representative of different settings (rural, suburban, and urban), and reflected common pathways for vaccination in China. Third, our study did not capture granular data on implementation and was not powered to test differences between the pay-it-forward and free-vaccine groups. Additional effectiveness research to compare different implementation strategies is needed to differentiate effective components and identify optimal pay-it-forward practices. Fourth, we recruited people who were already attending the clinics and it is likely that our participants might have better health literacy and behaviours than those who were not attending these clinics. However, we speculated that this bias might be similar across the two groups and unlikely to significantly affect the differences we observed between the two groups. Fifth, participants in the pay-it-forward group might have behaved differently knowing that they were being observed, affecting donating behaviours (defined as Hawthorne effect). However, whether a participant in the pay-it-forward group had donated money and how much they had donated were not revealed to local community members. As a result, we do not anticipate that there would have been a pronounced Hawthorne effect. In addition, since most people used WeChat to donate, the research assistants were not aware of a participant's donation status. Nevertheless, the psychological impact of research participation and the presence of researchers should be accounted for and examined in future implementation efforts. Sixth, our study included more women than men. This difference might be, partly, because we did not use quota sampling based on sex ratio but on a voluntary participation basis, and female participants are generally more responsive to research studies.^36^ Furthermore, women undertake more domestic work in the Chinese context and might be more likely to do childcare duties (ie, taking children to be vaccinated) than men.^37^ Finally, this was a quasi-experimental study and did not use randomisation. However, all standard-of-care periods were immediately followed by pay-it-forward periods (appendix p 7), decreasing the likelihood of temporal changes explaining the observed differences. In addition, our previous pay-it-forward quasi-experimental study results^21^ were similar to a subsequent randomised controlled trial.^22^

## Data sharing

Requests for data by researchers with proposed use of the data can be made to the corresponding author with specific data needs, analysis plans, and dissemination plans.

## Declaration of interests

We declare no competing interests.

## References

[1] Li L, Liu Y, Wu P (2019). Influenza-associated excess respiratory mortality in China, 2010–15: a population-based study. Lancet Public Health.

[2] Mameli C, Cocchi I, Fumagalli M, Zuccotti G (2019). Influenza vaccination: effectiveness, indications, and limits in the pediatric population. Front Pediatr.

[3] Conlon A, Ashur C, Washer L, Eagle KA, Hofmann Bowman MA (2021). Impact of the influenza vaccine on COVID-19 infection rates and severity. Am J Infect Control.

[4] Candelli M, Pignataro G, Torelli E (2021). Effect of influenza vaccine on COVID-19 mortality: a retrospective study. Intern Emerg Med.

[5] Principi N, Camilloni B, Esposito S, Group EVS (2018). Influenza immunization policies: which could be the main reasons for differences among countries?. Hum Vaccin Immunother.

[6] Wang Q, Yue N, Zheng M (2018). Influenza vaccination coverage of population and the factors influencing influenza vaccination in mainland China: a meta-analysis. Vaccine.

[7] Ortiz JR, Neuzil KM (2019). Influenza immunization in low- and middle-income countries: preparing for next-generation influenza vaccines. J Infect Dis.

[8] Wang J, Sun D, Abudusaimaiti X, Vermund SH, Li D, Hu Y (2019). Low awareness of influenza vaccination among pregnant women and their obstetricians: a population-based survey in Beijing, China. Hum Vaccin Immunother.

[9] Song Y, Zhang T, Chen L (2017). Increasing seasonal influenza vaccination among high risk groups in China: do community healthcare workers have a role to play?. Vaccine.

[10] Thomas RE, Lorenzetti DL (2018). Interventions to increase influenza vaccination rates of those 60 years and older in the community. Cochrane Database Syst Rev.

[11] US Department of Health and Human Services (June 2015). Principles of community engagement. 2nd edn. https://www.atsdr.cdc.gov/communityengagement/pdf/PCE_Report_508_FINAL.pdf.

[12] Tembo D, Hickey G, Montenegro C (2021). Effective engagement and involvement with community stakeholders in the co-production of global health research. BMJ.

[13] Li P, Hayat K, Jiang M (2021). Impact of video-led educational intervention on the uptake of influenza vaccine among adults aged 60 years and above in China: a study protocol for a randomized controlled trial. BMC Public Health.

[14] Liao Q, Fielding R, Cheung YTD, Lian J, Yuan J, Lam WWT (2020). Effectiveness and parental acceptability of social networking interventions for promoting seasonal influenza vaccination among young children: randomized controlled trial. J Med Internet Res.

[15] Tao L, Lu M, Wang X, Han X, Li S, Wang H (2019). The influence of a community intervention on influenza vaccination knowledge and behavior among diabetic patients. BMC Public Health.

[16] Lytras T, Kopsachilis F, Mouratidou E, Papamichail D, Bonovas S (2016). Interventions to increase seasonal influenza vaccine coverage in healthcare workers: a systematic review and meta-regression analysis. Hum Vaccin Immunother.

[17] Wong VW, Lok KY, Tarrant M (2016). Interventions to increase the uptake of seasonal influenza vaccination among pregnant women: a systematic review. Vaccine.

[18] Jiang X, Shang X, Lin J, Zhao Y, Wang W, Qiu Y (2021). Impacts of free vaccination policy and associated factors on influenza vaccination behavior of the elderly in China: a quasi-experimental study. Vaccine.

[19] (Dec 10, 2020). 2020 Guangzhou influenza vaccine price list. https://gz.bendizhidao.com/site/content/12578.html.

[20] Tang W, Wu D, Yang F (2021). How kindness can be contagious in healthcare. Nat Med.

[21] Li KT, Tang W, Wu D (2019). Pay-it-forward strategy to enhance uptake of dual gonorrhea and chlamydia testing among men who have sex with men in China: a pragmatic, quasi-experimental study. Lancet Infect Dis.

[22] Yang F, Zhang TP, Tang W (2020). Pay-it-forward gonorrhoea and chlamydia testing among men who have sex with men in china: a randomised controlled trial. Lancet Infect Dis.

[23] Li KT, Huang W, Tang W (2020). A secondary mixed methods analysis of a pay-it-forward gonorrhea/chlamydia testing program among men who have sex with men in China. Sex Transm Dis.

[25] Chinese Center for Disease Control and Prevention (2018). Technical guideline for influenza vaccination in China. http://www.chinacdc.cn/jkzt/crb/bl/lxxgm/jszl_2251/201809/t20180921_194050.html.

[26] Finley N, Swartz TH, Cao K, Tucker JD (2020). How to make your research jump off the page: co-creation to broaden public engagement in medical research. PLoS Med.

[27] Bolton WS, Ng S, Lam A (2021). Virtual hackathon to tackle COVID-19 unmet needs. BMJ Innov.

[28] Wei Z, Sun X, Yang Y, Zhan S, Fu C (2021). Seasonal influenza vaccine hesitancy profiles and determinants among Chinese children's guardians and the elderly. Expert Rev Vaccines.

[29] de Figueiredo A, Simas C, Karafillakis E, Paterson P, Larson HJ (2020). Mapping global trends in vaccine confidence and investigating barriers to vaccine uptake: a large-scale retrospective temporal modelling study. Lancet.

[30] Larson HJ, de Figueiredo A, Xiahong Z (2016). The state of vaccine confidence 2016: global insights through a 67-country survey. EBioMedicine.

[31] Anderson LJ, Shekelle P, Keeler E (2018). The cost of interventions to increase influenza vaccination: a systematic review. Am J Prev Med.

[32] Shobugawa Y, Fujiwara T, Tashiro A, Saito R, Kondo K (2018). Social participation and risk of influenza infection in older adults: a cross-sectional study. BMJ Open.

[33] Konrath S, Brown S (2013).

[34] Wang K, Wong ELY, Ho KF (2020). Intention of nurses to accept coronavirus disease 2019 vaccination and change of intention to accept seasonal influenza vaccination during the coronavirus disease 2019 pandemic: a cross-sectional survey. Vaccine.

[35] Del Riccio M, Lina B, Caini S (2021). Letter to the editor: increase of influenza vaccination coverage rates during the COVID-19 pandemic and implications for the upcoming influenza season in northern hemisphere countries and Australia. Euro Surveill.

[36] Smith WG (June, 2008). Does gender influence online survey participation?: a record-linkage analysis of university faculty online survey response behavior. https://files.eric.ed.gov/fulltext/ED501717.pdf.

[37] Yang J (2017). Gendered division of domestic work and willingness to have more children in China. Demogr Res.

