# Supplementary material for: Effectiveness of a pay-it-forward intervention compared with user-paid vaccination to improve influenza vaccine uptake and community engagement among children and older adults in China: a quasi-experimental pragmatic trial
Source: Lancet Infect Dis. 2022 Oct;22(10):1484–92. doi: 10.1016/S1473-3099(22)00346-2 (PMC9492551; doi:10.1016/S1473-3099(22)00346-2)
Supplement: Supplementary appendix [file mmc1.pdf]

THE LANCET
Infectious Diseases

Supplementary appendix

This appendix formed part of the original submission and has been peer reviewed. We post it as supplied by the authors.

Supplement to: Wu D, Jin C, Bessame K, et al. Effectiveness of a pay-it-forward intervention compared with user-paid vaccination to improve influenza vaccine uptake and community engagement among children and older adults in China: a quasi-experimental pragmatic trial. *Lancet Infect Dis* 2022; published online July 19. https://doi.org/10.1016/S1473-3099(22)00346-2.

SUPPLEMENTARY VIDEO LINK HERE.

The video demonstrates an older Chinese lady who received a vaccination through our pay-it-forward project and contributed to developing this video in order to illustrate the pay-it-forward concept for further communication purposes. The video script was co-developed and edited by an older lady as a community representative who is aged above 70 years old and from Guangzhou, the capital city of Guangdong province. The lady worked with the team on the procedures of receiving a vaccination at a clinic of a medical center and acted in the video.

SUPPLEMENTARY FIG 1: PAY-IT-FORWARD MODEL OVERVIEW

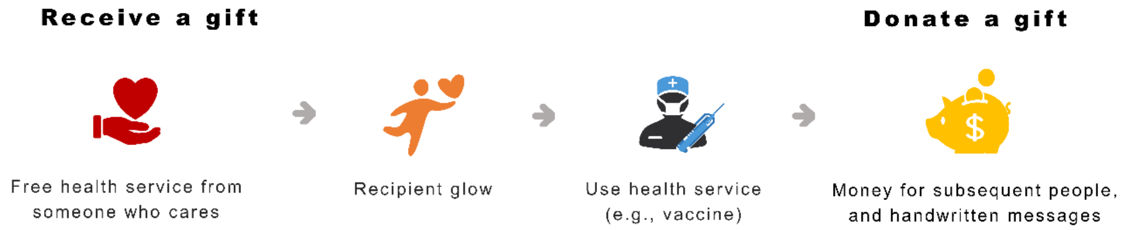

COMMUNITY ENGAGEMENT SECTION

Part A: Behavioral mechanisms

We discussed possible economic-behavioral or psycho-social mechanisms that can explain the success of pay-it-forward in our internal meetings. One commonly used theory is generalized reciprocity that suggests that people who have received help are more likely to help a third party.[1,2] How people perceive others' kindness can potentially change personal donations.[3] Additionally, pay-it-forward has two quite different features – receiving a gift and giving a gift. Jung et al concluded that when people are reminded of giving rather than receiving a gift, they would pay forward and with a higher amount.[3]

Part B: Donation strategies

The donation strategies were developed iteratively. We field tested several donation strategies (see below) to increase the donation amount.

1) a closed ended survey question asking about willingness to donate (yes/no), followed by an open ended question to indicate donation amount.

2) a closed ended survey question asking about willingness to donate (yes/no), followed by a closed ended question asking about donation amount with options in descending order (CNY200, 150, 100, 50, and another amount).

3) a closed ended survey question asking about willingness to donate (yes/no), followed by a closed ended question asking about donation amount with options in descending order with indication of the additional number of people that the respondent would support by donating that amount (CNY200 that can support one older person and 1 child, CNY150 that can support 1 older people or 3 children, CNY100 that can support 2 children, CNY50 that can support 1 child, and another amount).

We found that strategy number 3 generated the highest donation amounts and hence adopted this donation strategy for the formal study. Many people chose to support 1 older people or a child.

Additionally, we asked whether participants were willing to donate money prior, not after, receiving vaccination because of several practical considerations. First and foremost, considering possible risks of COVID, we tried to minimize direct contacts between research assistants and participants during

the process via completing the research process at one meeting (i.e., project introduction, consenting, questionnaire administration, and donations) and research staff did not need to make further in-person communications after the participants were directed to clinical staff for receiving the vaccination. Second, after being given information about pay-it-forward and a free influenza vaccine from people who care, making a donation decision tends to be a rather intuitive and quick decision. Therefore, providing channels for receiving a donation right after participants' completion of the survey could facilitate translating the intention to act and also reduce the possibility of losing potential donors due to lack of donation mechanisms. But for some cases who wanted more time to make a decision on donation, we made it clear that they could decide whether they wanted to donate after receiving the vaccination. Lastly, there is a chance that people might simply forget about the project after a series of clinical process at the clinic or did not have time to come back to our research staff to make the donation. In summary, we believe providing an opportunity for participants to make an immediate donation after the project introduction is an optimal solution.

Part C: Community engagement strategies

Community engagement strategies used during the study implementation in Guangdong China included the following: inviting community members to co-design postcards based on the conceptual ideas mapped out during the hackathon (supplementary file pages 2-7: community engagement section Part C); working in partnership with a local older adult to co-develop a video to explain pay-it-forward (supplementary file page 1: video link); inviting study participants to write postcard messages during recruitment for future participants (hachathon outputs section Part A in supplement); and engaging local medical staff in implementing the quasi-experimental study, including having one-to-two staff members at each study site to help adjust recruitment and communication efforts to the local dialect. The community's six postcard designs were subsequently used to explain the pay-it-forward system to potential participants.

Part D: Design of postcards and messages

- **Postcard design conceptual ideas mapped out during the hackathon event**

Note: These design concepts took the Chinese culture into account and were developed by three Chinese attendees and one Amrican father of Chinese children.

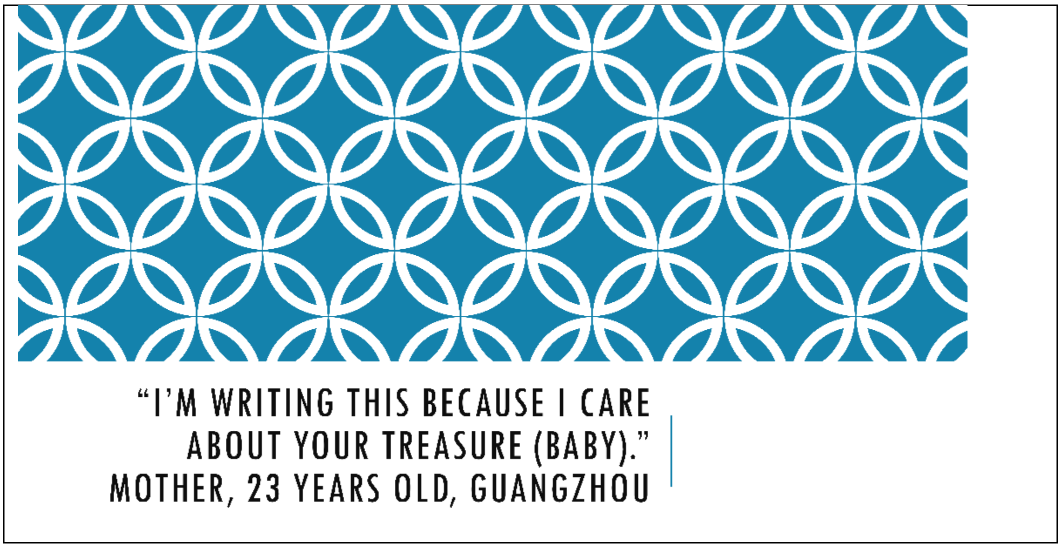

[BACK OF POSTCARD]

Every year in China, 19,000 people die from influenza. This preventable infectious disease disproportionately affects babies and grandparents. Influenza vaccines prevent the disease and save lives. The vaccine is easy to get, reliable, recommended by public health authorities, and safe. Millions of people receive this vaccination every year.

Today another parent in Guangzhou has donated the money for your child to receive a vaccine. This is part of a pay-it-forward project. Pay-it-forward has one person receive a free vaccine and then choose whether they want to donate for the next person's child to receive a vaccine.

[infographic on influenza vaccination]

"A chance to go to a top school.

An apartment after you graduate.

You'd do almost anything for your kids. Why not vaccinate them against influenza?"

-Father, 34 years old, Guangzhou

OPPORTUNITY COST

Every time your kid gets the flu, you lose important time at your work, your kid misses school lessons, your grandparents have added pressure, and the whole family is at increased risk of flu. Why not get vaccinated against influenza today?

-Father, 22 years old, Guangzhou

FILIAL PIETY

- Help grandparents with heavy bags.
- Obey your parents.
- Vaccinate your child.

GRANDPARENT BENEFIT QUOTE

"Vaccinating your child against influenza decrease the risk of maternal grandma, maternal grandpa, paternal grandma, and paternal grandpa from getting the influenza which can be deadly among the elderly. Did you know that XX grandparents die from influenza each year?"

Not vaccinating your child is disrespects the elderly. Vaccinating your child against influenza decrease the risk of maternal grandma, maternal grandpa, paternal grandma, and paternal grandpa from getting the influenza which can be deadly among the elderly. Did you know that XX elderly die from influenza each year?

- **Community co-designed postcards**

Note: This series of postcards were co-designed by three community representatives with backgrounds in communication and social media. All three of them were women aged between 27 and 34. These postcards were developed and refined based on the postcard design conceptual ideas we mapped out during the hackathon event.

Postcard images	Cover messages
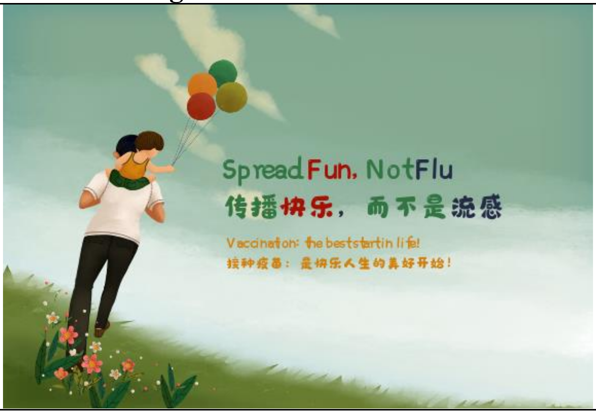	Spread fun, not flu.<br>Vaccination is a beautiful start of one's life.

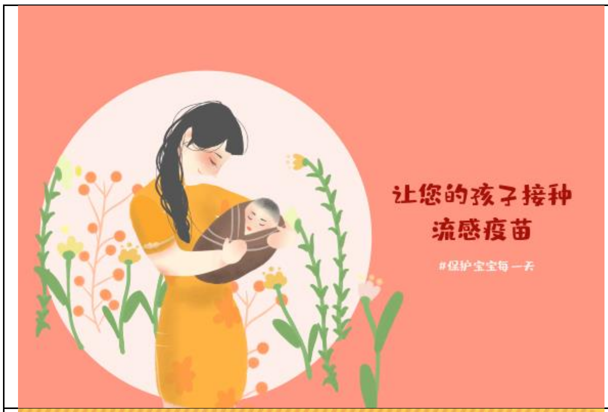	Vaccinate your child against influenza.<br>#ProtectYourBabyEveryday
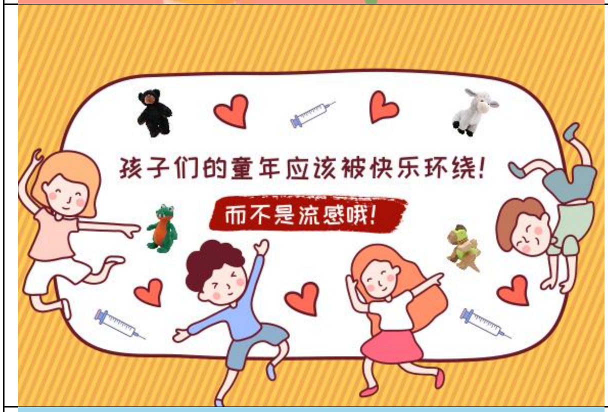	Children should be surrounded by fun, not flu.
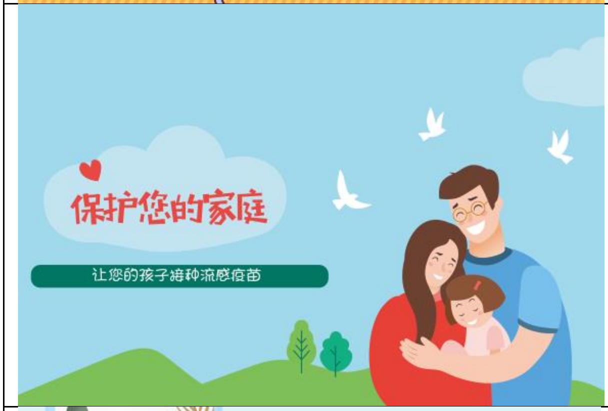	Vaccinate your child and protect your family
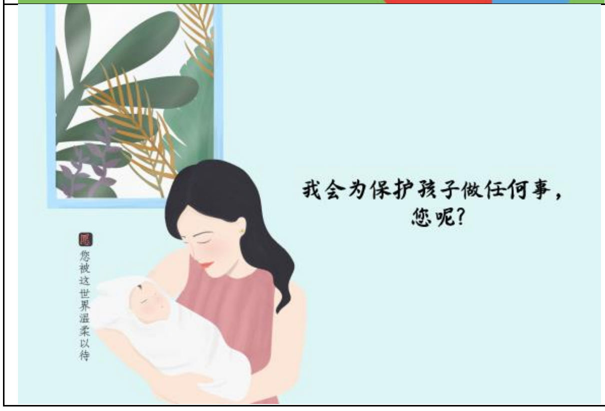	I'll do anything to protect my kids. Will you?

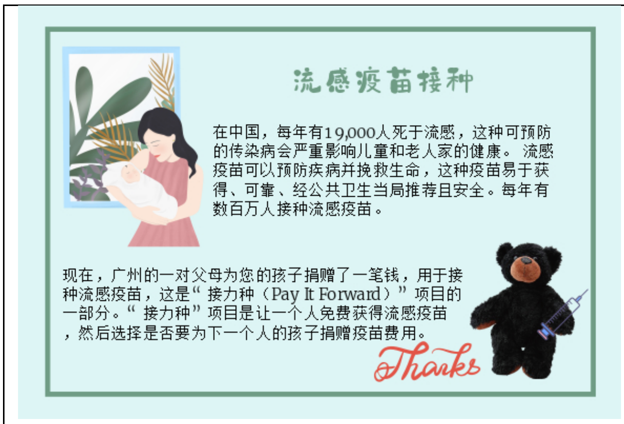	Every year in China, 19,000 people die from influenza. This preventable infectious disease disproportionately affects babies and grandparents. Influenza vaccines prevent the disease and save lives. The vaccine is easy to get, reliable, recommended by public health authorities, and safe. Millions of people receive this vaccination every year.<br>Today another parent in Guangzhou has donated the money for your child to receive a vaccine. This is part of a pay-it-forward project. Pay-it-forward has one person receive a free vaccine and then choose whether they want to donate for the next person's child to receive a vaccine.

- **Translated hand-written postcard messages from the participants.**

Note: These handwritten postcard card messages were delivering good wishes to strangers and some of them were relevant to encouraging other to vaccinate.

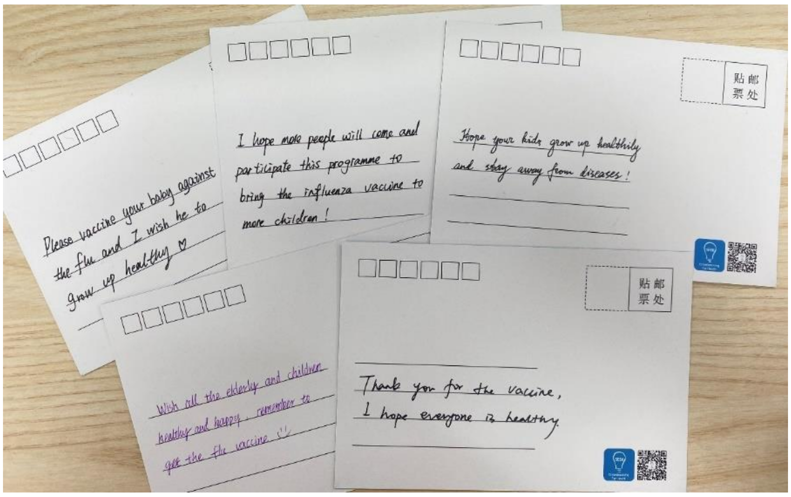

SUPPLEMENTARY FIG 2 TIME-BASED RECRUITMENT FOR THE THREE STUDY ARMS AT THREE STUDY SITES

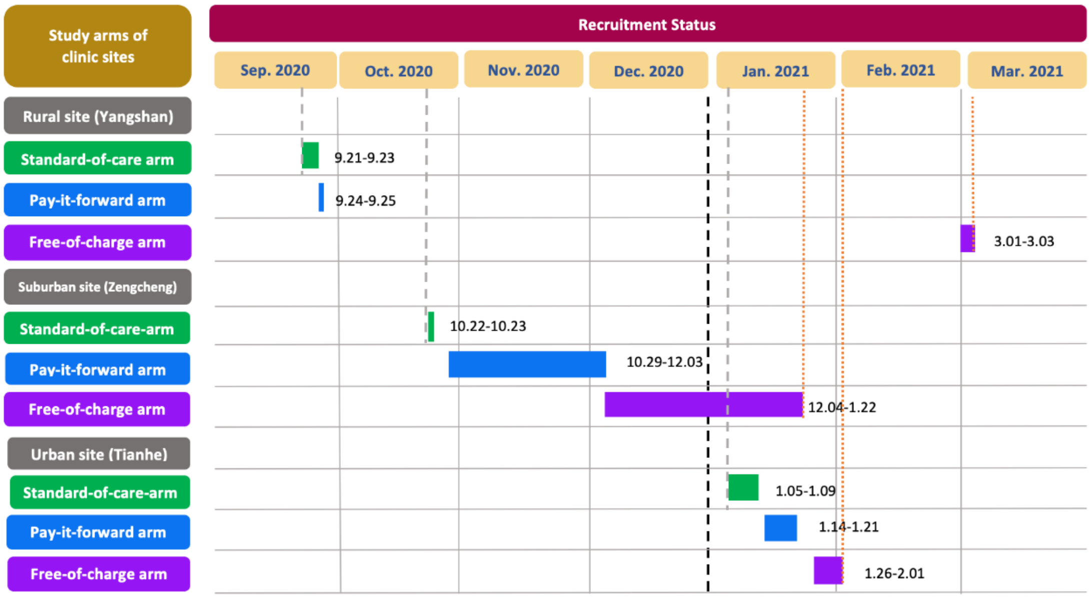

* Each site only used one arm at a single time period and all three sites had standard-of-care arm followed directly by pay-if-forward arm.

Footnote: The recruitment in Yangshan was spaced out because of occasional stock outs and COVID vaccination. The lengths of time for the three arms at the study sites varied due to variations in patient volume and recruitment pace.

SUPPLEMENTARY FIG 3: INTRODUCTION TO INFLUENZA (ENGLISH TRANSLATION).

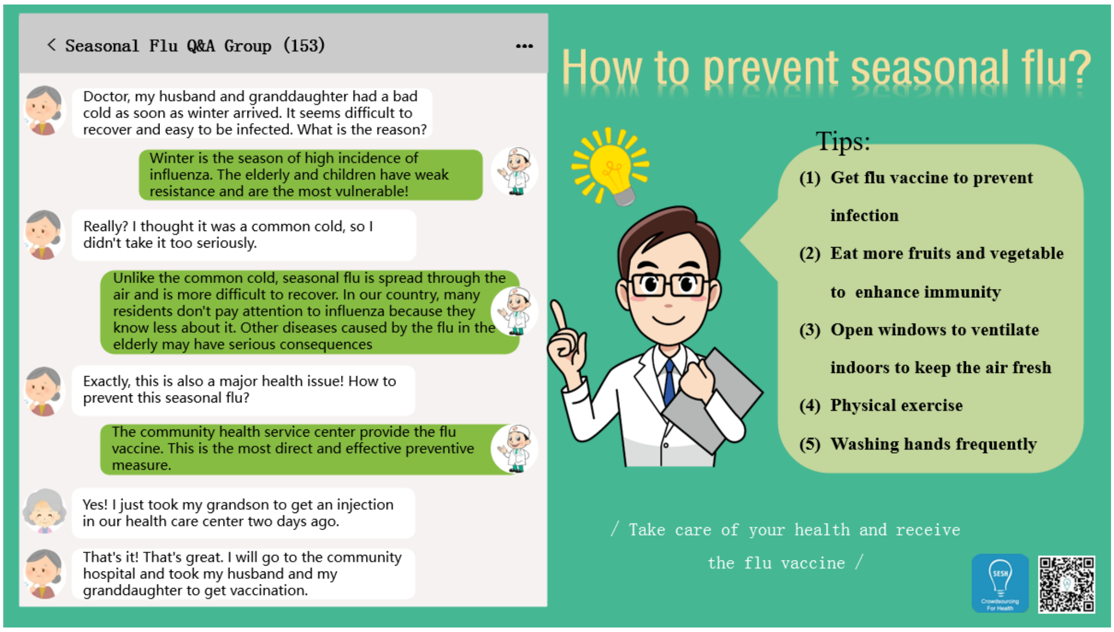

SUPPLEMENTARY FIG 4: PAY-IT-FORWARD PAMPHLET (ENGLISH TRANSLATION).

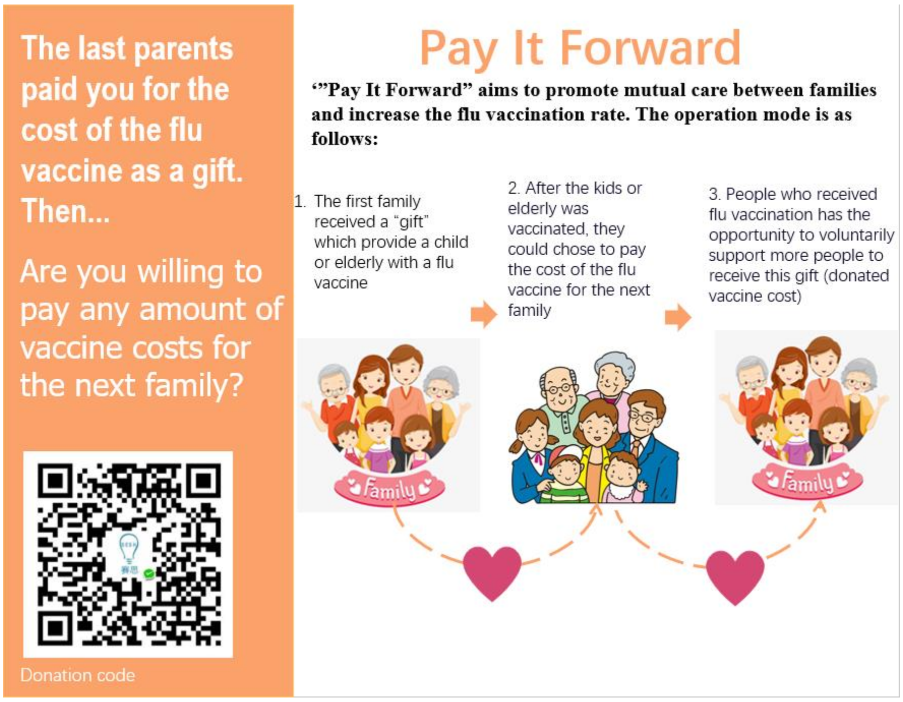

SUPPLEMENTARY FIG 5: VACCINE UPTAKE IN STANDARD-OF-CARE AND PAY-IT-FORWARD ARMS BY STUDY SITES AMONG THE CHILD GROUP

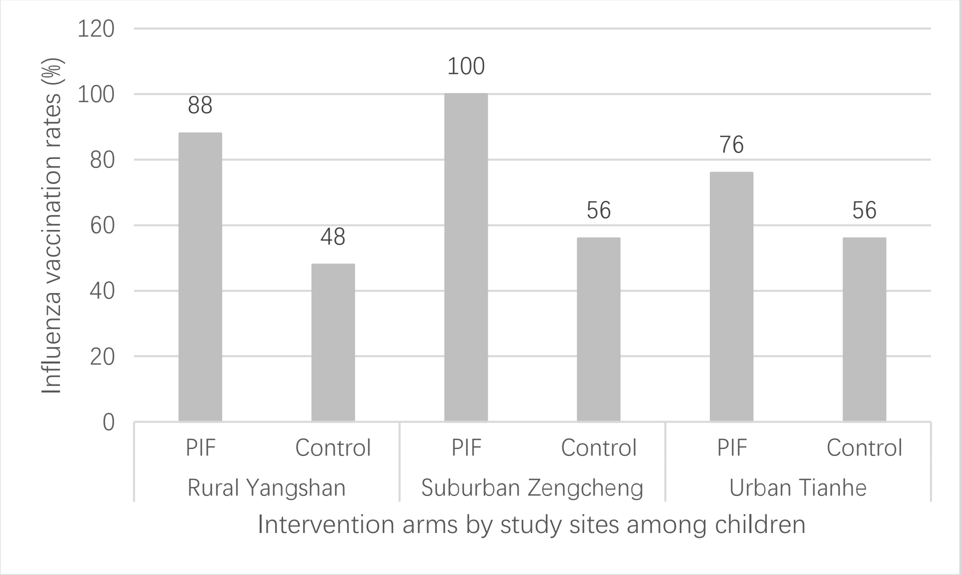

SUPPLEMENTARY FIG 6: VACCINE UPTAKE IN STANDARD-OF-CARE AND PAY-IT-FORWARD ARMS BY STUDY SITES AMONG THE OLDER GROUP

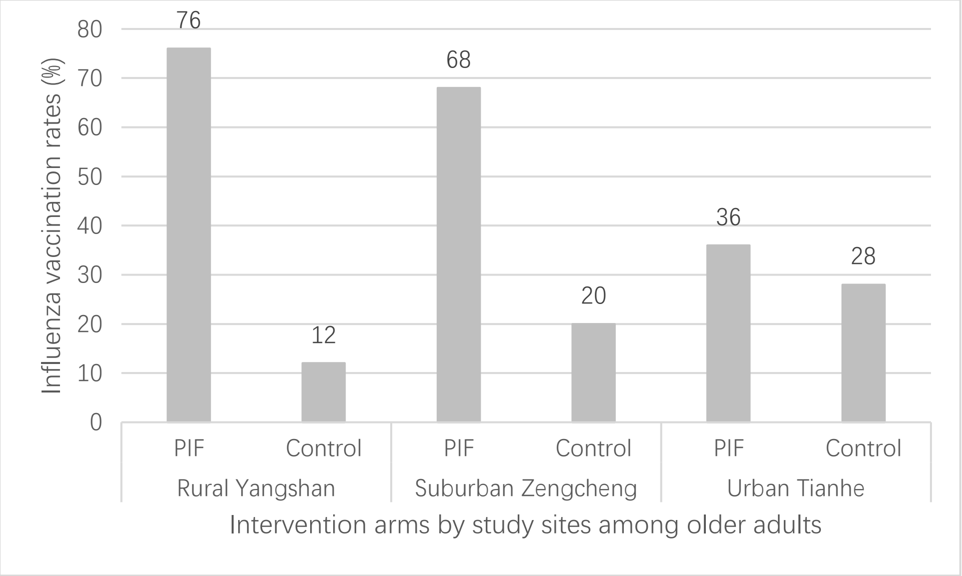

SUPPLEMENTARY FIG 7: DISTRIBUTION OF DONATION AMOUNTS BY STUDY SITE (N=107)

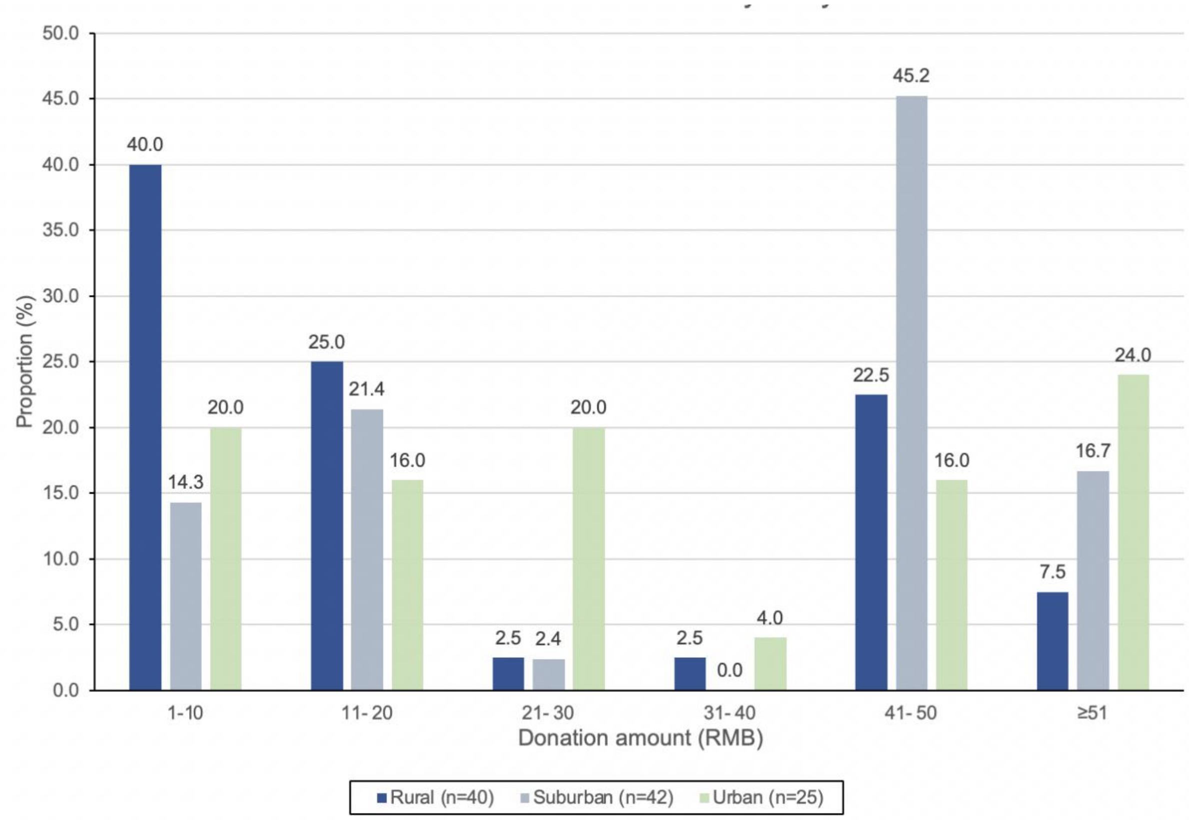

Note: 1 US$=6.59 RMB

SUPPLEMENTARY FIGURE 8: PERCENTAGES OF DONATIONS OF VACCINE COSTS BY STUDY SITES AND AGE GROUP, N=107.

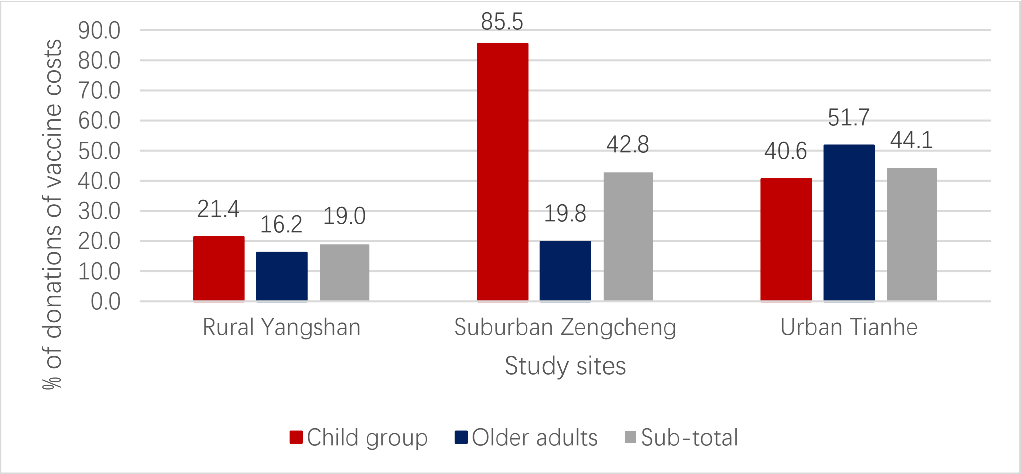

SUPPLEMENTARY TABLE 1: PROPORTION DIFFERENCES IN INFLUENZA VACCINATION RATES BETWEEN THE PAY-IT-FORWARD AND STANDARD-OF-CARE ARMS BY AGE GROUPS IN GUANGDONG PROVINCE, CHINA, 2020-2021 (N=300)

	Children<br>N=150		Older adults<br>N=150	
**Uptake (n,%)**		**P value**<br>**<0.0001**		**P value**<br>**<0.0001**
Standard of care	40 (53.3)		15 (20)	
Pay-it-forward	66 (88)		45 (60)	
**Study arms**	**Crude proportion difference (95%CI)**	**P value**	**Crude proportion difference (95%CI)**	**P value**
Pay-it-forward vs Standard of care	34.7 (21.2, 48.1)	<0.0001	40.0 (25.7, 54.3)	<0.0001
**Adjusting study site**	**Adjusted proportion difference (95%CI)**	**P value**	**Adjusted proportion difference (95%CI)**	**P value**
Pay-it-forward vs Standard of care	34.7 (21.4, 47.9)	<0.0001	40.0 (26.3, 53.7)	<0.0001
**Adjusting education level**				
Pay-it-forward vs Standard of care	36.3 (22.1, 50.5)	<0.0001	41.1 (27.1, 55.1)	<0.0001

SUPPLEMENTARY TABLE 2: DISTRIBUTION OF DONATION STATUS AND PARTICIPANTS WITH A DONATION OF 50 RMB OR ABOVE BY STUDY SITE, N(%)

**Characteristic**	Rural (n=50)	Suburban (n=50)	Urban (n=50)	p-value*
**Donation status (n=150)**				
- *Donated*	40 (80.0)	42 (84.0)	25 (50.0)	<0.001
- *Did not donate*	10 (20.0)	8 (16.0)	25 (50.0)	
**Characteristic**	Rural (n=40)	Suburban (n=42)	Urban (n=25)	p-value*
**Donation amount (n=107)**				
- *<7.59 USD*	28 (70.0)	16 (38.1)	15 (60.0)	0.013
- *>=7.59 USD*	12 (30.0)	26 (61.9)	10 (40.0)	

SUPPLEMENTARY COSTS FILE

We compared costs between all three arms. We adopted the same sample size of 150 (75 children and 75 older adults) for the free arm as the other two arms. Participants in the free vaccination arm were invited to participate using the same introductory pamphlet and were provided with free influenza vaccination. They did not receive any community-created messages about the pay-it-forward program. After recruiting all participants for the free arm, we collected costs data (cost file table 1) and analyzed costs (costs file figure 1 and table 2). Economic and financial costs per person are reported in the main text.

Costs file Table 1: *Unit costs (in 2020 USD) and frequency of vaccine use*

Intervention	Cost item	Unit cost	Resource use	Source
Pay-it-forward		Staff wage per hour (USD/hour)*		
• Start-up costs				
	Time designing postcards to be written on by participants in the PIF programme†	7.47	1 x 5 hr	Personal communication with research staff
	Time of research fellow participating in preparatory workshop†	12.7	1 x 5 hr	Personal communication with research staff
	Time of research assistant participating in preparatory workshop†	7.47	1 x 5 hr	Personal communication with research staff
	Time of nurses participating in preparatory workshop	4.81	3 x 1 hr	Personal communication with research staff<br>China Social Welfare Foundation [4]
	Time of clinic coordinators participating in preparatory workshop	4.81	3 x 1 hr	Personal communication with research staff China Social Welfare Foundation[4]

• Fixed costs				
	Vaccinators (doctors) for the three clinics	11.07	3 x 70 hr	China Social Welfare Foundation [4]
• Recurrent costs				
	Time of nurses in recruiting patients to join the PIF programme††	4.81	3 x 25 hr	Personal communication with research staff<br>China Social Welfare Foundation [4]
	Time of clinic coordinators in performing administrative work for the PIF programme††	4.81	3 x 25 hr	Personal communication with research staff<br>China Social Welfare Foundation [4]
		Cost per vaccine (USD)		
	Cost of adult vaccines in Yangshan#	22.9	41	Health clinic reimbursement invoices
	Cost of adult vaccine in Zengcheng	22.9	17	Health clinic reimbursement invoices
	Cost of child vaccine in Zengcheng	8.38	25	Health clinic reimbursement invoices
	Cost of adult vaccine in Tianhe#	12.2	28	Health clinic reimbursement invoices
		Cost per item / batch (USD)		

	Introductory pamphlets for the PIF programme (batch cost)	29.0	1	Project research staff invoices
	Ballpoint pens (for writing messages on PIF postcards) (batch cost)	2.25	1	Project research staff invoices
	Cost of printing postcards (batch cost)	7.41	1	Project research staff invoices
	Surgical gloves	0.075	222	Personal communication with research staff

**Intervention**	**Cost item**	**Unit cost**	**Resource use**	**Source**
Standard-of-care		Staff wage per hour (USD/hour)*		
• Start-up costs	*Time of nurses participating in preparatory workshop*	4.81	3 x 1 hr	China Social Welfare Foundation [4]
	Time of clinic coordinators participating in preparatory workshop	4.81	3 x 1 hr	China Social Welfare Foundation [4]
• Fixed costs				
	Vaccinators for the three clinics	11.07	3 x 70 hr	China Social Welfare Foundation [4]
• Recurrent costs				
	Time of nurses in recruiting patients to receive seasonal influenza vaccination††	4.81	3 x 12.5 hr	Personal communication with research staff

				China Social Welfare Foundation [4]
	Time of clinic coordinators in performing administrative work for influenza vaccination††	4.81	3 x 12.5 hr	Personal communication with research staff<br>China Social Welfare Foundation [4]
		Cost per vaccine (USD)		
	Cost of adult vaccines in Yangshan#	22.9	15	Health clinic reimbursement invoices
	Cost of adult vaccine in Zengcheng	22.9	5	Health clinic reimbursement invoices
	Cost of child vaccine in Zengcheng	8.38	14	Health clinic reimbursement invoices
	Cost of adult vaccine in Tianhe#	12.2	21	Health clinic reimbursement invoices
		Cost per item (USD)		
	Surgical gloves	0.075	110	Personal communication with research staff
**Intervention**	**Cost item**	**Unit cost**	**Resource use**	**Source**
Free vaccination				
• Start-up costs		Staff wage per hour (USD/hour)*		
	Time of nurses participating in preparatory workshop	4.81	3 x 1 hr	Personal communication with research staff<br>China Social Welfare Foundation [4]
	Time of clinic coordinators participating in preparatory workshop	4.81	3 x 1 hr	Personal communication with research staff<br>China Social Welfare Foundation [4]

• Fixed costs			
Vaccinators for the three clinics	11.07	3 x 70 hr	China Social Welfare Foundation [4]
• Recurrent costs			
Time of nurses in recruiting patients to join the PIF programme††	4.81	3 x 12.5 hr	Personal communication with research staff<br>China Social Welfare Foundation [4]
Time of clinic coordinators in performing administrative work for the PIF programme††	4.81	3 x 12.5 hr	Personal communication with research staff<br>China Social Welfare Foundation [4]
	Cost per vaccine (USD)		
Cost of adult vaccines in Yangshan#	22.9	42	Health clinic reimbursement invoices
Cost of adult vaccine in Zengcheng	22.9	13	Health clinic reimbursement invoices
Cost of child vaccine in Zengcheng	8.38	18	Health clinic reimbursement invoices
Cost of adult vaccine in Tianhe#	12.2	41	Health clinic reimbursement invoices
	Cost per item / batch (USD)		
Surgical gloves	0.075	228	Personal communication with research staff
Information pamphlet for free vaccination arm	20.4	1	Project research staff invoices

PIF *- pay-it-forward,* ***USD*** *– United States dollars*

**Hourly wages were calculated from monthly or yearly wages based on an assumption of 250 working days per year and 10 working hours per day.*

†These costs were annualised over a three-year period at a discount rate of 3% as they were part of the preparatory workshop for the conception of PIF programme specifically. The training, supplies and expertise gained through these sessions are expected to be useful in future years to inform further iterations of the programme and were therefore annualised over three years.

†† It was assumed that each nurse spent 10 minutes per patient to recruit and persuade participants to be vaccinated in the pay-it-forward programme, while they spent 5 minutes per patient for the standard-of-care group, and 5 minutes per patient in the free vaccination arm.
#Only adult influenza vaccine was used in Yangshan and Tianhe, i.e. children were inoculated with the same influenza vaccine as the adults in those study sites.

Addendum: Breakdown for obtaining the total economic cost and the total financial cost of each intervention arm

Total economic cost of an intervention
= Start-up costs + Fixed costs + Recurrent costs

Total financial cost of an intervention
= Start-up costs + Fixed costs + Recurrent costs – (Payment / Donation contributions)

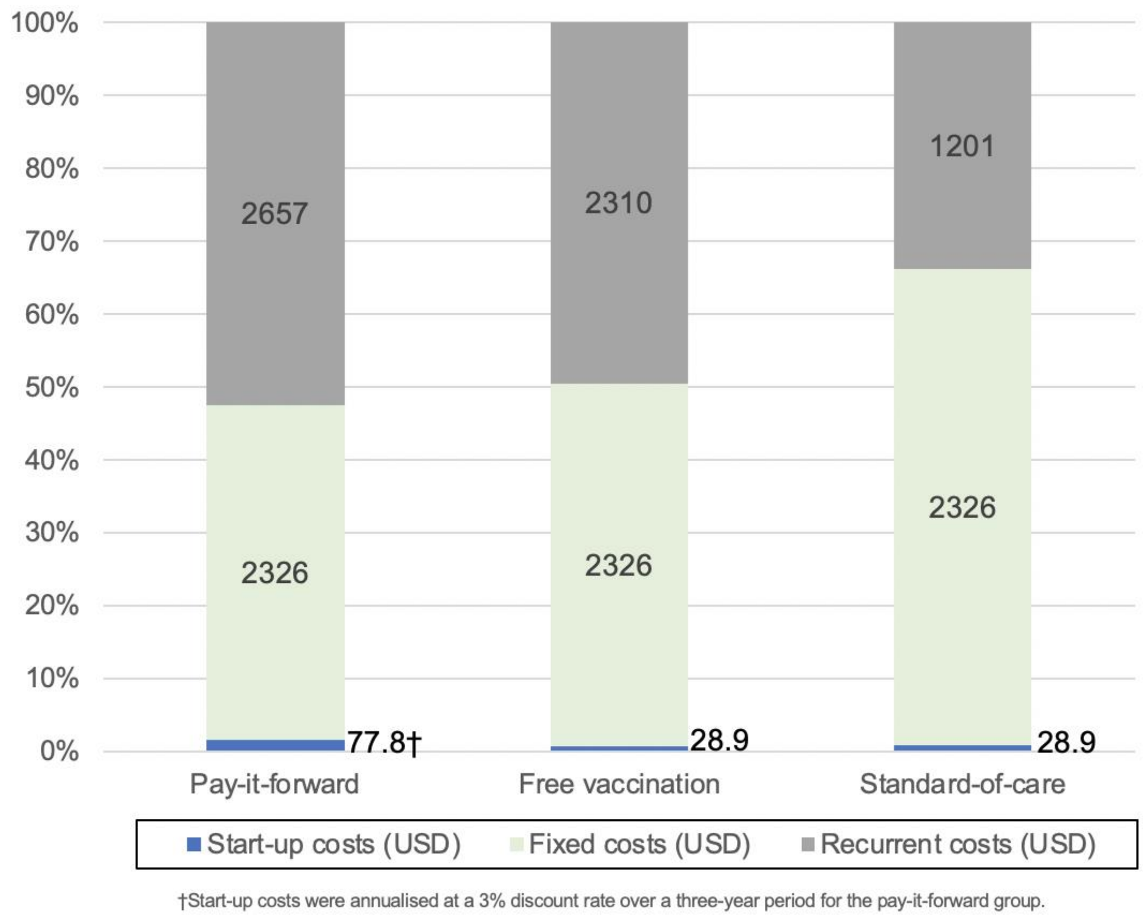

Costs file Figure 1: Breakdown of economic costs by category in the three intervention arms

Costs file *Table 2: Total economic costs and total financial costs by intervention arm*

Intervention arm	Total economic cost (USD)	Total financial cost (USD)
Pay-it-forward	5062	4477
Free vaccination	4665	4665
Standard-of-care	3557	2725

Note: See Table 1 for detailed breakdown of the economic and financial costs

SUPPLEMENTARY QUESTIONNAIRE

Influenza vaccination for children and Older people

About this study:

You are invited to participate in this influenza vaccination study. The results of this study can provide evidence for the government to develop better intervention strategies to promote influenza vaccination in China and prevent seasonal influenza among at-risk populations.

Details of this study:

You will need to fill out a questionnaire. This questionnaire will ask about your socio-demographic information and opinions towards influenza vaccine. To protect your privacy, all your answers will be transformed into digital formats and password encrypted. If you are eligible to participate in this program, out project personnel will provide you with more detailed information. To confirm whether you are qualified to participate in this program, we will first ask you a few screening questions. If you are not eligible for this program, we will not keep your survey information.

Note: If the participant is a child, the guardian will need to consent on the child's behalf and fill in the questionnaire. For the purpose of equality, only one quota is provided for each participating family, either for a child or an Older individual.

Screening questions (all groups)

i.Which category do you belong to____

○Caregivers of a 3-8 year-old children

○Older people (>=60)

ii. Your age (fill in a number) ____

iii. Your gender（male-1, female-2） ___

iv. Have you been vaccinated against influenza in the past year?

○Yes (Please skip to the end of the questionnaire and submit the answer sheet)

○No

A. Basic information (all groups)

1. Ethnicity

○Han

○Other, please specify： ____________

2. Your highest education level:

○Primary school
○Junior middle school

○High school

○Undergraduate or college

○Postgraduate or above

3. Your occupation is:

○Student

○Civil servant

○Farmer

○Ordinary workers (blue collar)

○Company staff (white collar)

○Technical personnel

○Unemployed or retired

○Other： ____________________*

4. Your personal monthly income:

○0-1000 RMB/Month

○1000-5000 RMB/Month

○5000-10,000 RMB/Month

○10,000 RMB/Month or above

5. Your current legal marriage status is:

○Unmarried

○Engaged or married

○Separated or divorced

○Widowed (skip questions 6-9)

6. Age of your partner： ____

7. The highest education level of your partner:

○Primary school

○Junior middle school

○High school

○Undergraduate or college

○Postgraduate

8. The occupation of your partner is:

○Student

○Civil servant

○Farmer

○Ordinary workers (blue collar)

○Company staff (white collar)

○Technical personnel

○Unemployed or retired

○Other： __________________*

9. Your partner’s personal monthly income

○0-1000 RMB/Month

○1000-5000 RMB/Month

○5000-10,000 RMB/Month

○10,000 RMB/Month or above

10 Do you have children in your family?

○Yes

○No (skip question16)

11. How many children are there in your family? (fill in your children's age and sex in chronological order, for example, Child 1, 2 years old, male-1 or female-2)

○Child 1

Age_____

Sex_____

○Child 2

Age_____

Sex_____

○Child 3

Age_____

Sex______

○Child 2

Age_____

Sex_____

○Child 2

Age_____

Sex_____

12. Do you have older people in your family?

○Yes

○No

13. How many older people (>=60 years) are there in your family? Age and gender?

○Older individual 1

Age:

Sex:

○Older individual 2

Age:

Sex:

B-0. “Standard of care” group

1. Main purpose of coming to the clinic？

○See a doctor for myself

○Take my child(ren) to see a doctor

○Take my child(ren) to vaccinate

○Accompany older family members to see a doctor

○ Health check-up

○Other： ____________________ *

2. The researcher has introduced the influenza vaccine. Would you like to participate in the program to vaccinate your child (or yourself if older than 60 years) today?

○Yes

○No（Please skip to the question 22）

3. Who would you like to vaccinate today?

○My Child

○An older family member

4. Information about the individual who receives the vaccination

○Sex___

○Age___

5. Has anyone in your family got seasonal flu (or flu-like illnesses) in the past year?

○Yes

○No（Skip to the Question 24）

6.If yes, who got seasonal flu (or flu-like illnesses) in your family?

○Children

○Older people

○Children’s parents

7. If you want your family to vaccinate, what is the main reason?

○Children in my family are easy to get seasonable flu

○Older people in my family are easy to get seasonable flu

○Both my children and older people in my family are easy to get seasonable flu

○I’m easy to get seasonable flu

○Recommended by my friends/family member

○Recommendation by healthcare practitioners at the clinic

○Other, please note： ______________ *

8. If you don't want to vaccinate your child or an Older individual in your family today, why not? [multiple choices] * (if you answered "yes" to question 2, skip this item)

□I don't know enough about seasonal influenza

□I'm not sure about the effect of the influenza vaccine

□My family don't need vaccinations

□It’s too cumbersome

□I'm worried about the side effects

□ I think it is too expensive

□Other, please specify: _______________*

233 **B-1. “Pay it forward” group**

1. Main purpose of coming to the clinic？

○See a doctor for myself

○Take my child(ren) to see a doctor

○Take my child(ren) to vaccinate

○Accompany older family members to see a doctor

○Other：____________________ *

2. The researcher has introduced the influenza vaccine and 'Pay it forward' program to you. Would you like to participate in the program to vaccinate your child (or yourself if older than 60 years) today?

○Yes

○No（Please skip to the question 27）

3. Who would you like to vaccinate today?

○My Child

○Older family member

4. Information about the individual who receives the vaccination

○Sex___

○Age___

5. Are you willing to donate some money to the next family to get the same influenza vaccination?

○Yes

○No（Skip to the Question 25）

6. How much would you like to donate to the next family?

○200 RMB (support 1 older individual + 1 child)

○150 RMB (support 1 older or 3 children)

○100 RMB (support 2 children)

○50 RMB (support 1 child)

○Other amount_____

7. If you want your family to vaccinate, what is the main reason?

○Children in my family are easy to get seasonable flu

○Older people in my family are easy to get seasonable flu

○Both my children and the old people in my family are easy to get seasonable flu

○I’m easy to get seasonable flu

○Recommended by my friends/family member

○Recommendation by medical personnel at the clinic (irrelevant to ‘Pay it forward’ program)

○The ‘Pay it forward’ program

○Other, please note： ______________ *

8. What do you think are the benefits of the ‘Pay it forward’ program?

[Multiple choice]

□Other families’ donation can lower my financial burden

□I learn about influenza vaccines that can prevent flus

□Can promote more families to be vaccinated against influenza

□Can reduce the spread of influenza

□Spread love and warmth within the community

□Other： _______________________ *

9. If you don't want to vaccinate your child or an Older individual in your family today, why not? [multiple choices] * (if you answered "yes" to question 2, skip this item)

□I don't know enough about seasonal influenza

□I'm not sure about the effect of the influenza vaccine

□My family don't need vaccinations

□It’s too cumbersome

□I'm worried about the side effects

□Other, please specify: _______________*

C. Vaccine related information (all groups)

1. Have you heard about the flu vaccine before this program?

○Yes ○No

2. In general, I think influenza vaccine is important. [Single choice] *

○Strong disagree ○Disagree ○Agree ○Strongly agree

3. In general, I think the influenza vaccine is safe. [Single choice] *

○Strong disagree ○Disagree ○Agree ○Strongly agree

4. In general, I think the influenza vaccine is effective. [Single choice] *

○Strong disagree ○Disagree ○Agree ○Strongly agree

5. Has your child ever been vaccinated against influenza?

(if there is no child in your family, please skip this question) [Single choice] *

○Yes ○No

6. Have older people in your family ever been vaccinated against influenza

(if there are no older individuals in your family, please skip this question) [Single choice] *

○Yes ○No

7. Have you ever been hesitant about having your child or older family members to get the flu vaccine (except for allergies)? [Single choice] *

○Yes ○No

8. Have you ever postponed your child or older family members to get the flu vaccine (except for allergies)? [Single choice] *

○Yes ○No

9. Have you ever been refused to have your child or older family members to get the flu vaccine (except for allergies)? [Single choice] *

○Yes ○No

10. Have you ever heard about negative information about influenza vaccine?

○Yes ○No

11. Do any of your friends or relatives object to the influenza vaccination?

○Yes ○No

12. Have you or people around you had adverse reactions to influenza vaccination?

○Yes ○No

13. Do you trust the advice provided by the medical personnel in the clinic on influenza vaccine?

○Yes ○No

14. Is price of the vaccine a barrier for your child and/or older individuals in your family to get the influenza vaccine?

○Yes ○No

1. Chuan A, Zhang HJAaS. Generalized Reciprocity: Theory and Experiment. 2021.
2. Gray K, Ward AF, Norton MI. Paying it forward: Generalized reciprocity and the limits of generosity. *Journal of experimental psychology: General* 2014; **143**(1): 247.
3. Jung MH, Nelson LD, Gneezy A, Gneezy U. Paying more when paying for others. *J Pers Soc Psychol* 2014; **107**(3): 414-31.
4. China Social Welfare Foundation 919 Nurse Care Scheme. 2017. Chinese Nurse Group Development Situational Report. (中國護士群體發展現狀調查報告).
